# PSD95 Suppresses Dendritic Arbor Development in Mature Hippocampal Neurons by Occluding the Clustering of NR2B-NMDA Receptors

**DOI:** 10.1371/journal.pone.0094037

**Published:** 2014-04-04

**Authors:** Fernando J. Bustos, Lorena Varela-Nallar, Matias Campos, Berta Henriquez, Marnie Phillips, Carlos Opazo, Luis G. Aguayo, Martin Montecino, Martha Constantine-Paton, Nibaldo C. Inestrosa, Brigitte van Zundert

**Affiliations:** 1 Center for Biomedical Research, Faculty of Biological Sciences and Faculty of Medicine, Universidad Andres Bello, Santiago, Chile; 2 Faculty of Biological Science, Universidad de Concepción, Concepción, Chile; 3 Department of Molecular and Cellular Biology, Faculty of Biological Sciences, Pontificia Universidad Católica de Chile, Santiago, Chile; 4 McGovern Institute for Brain Research, Massachusetts Institute of Technology, Cambridge, Massachusetts, United States of America; 5 FONDAP Center for Genome Regulation, Santiago, Chile; University of Michigan, United States of America

## Abstract

Considerable evidence indicates that the NMDA receptor (NMDAR) subunits NR2A and NR2B are critical mediators of synaptic plasticity and dendritogenesis; however, how they differentially regulate these processes is unclear. Here we investigate the roles of the NR2A and NR2B subunits, and of their scaffolding proteins PSD-95 and SAP102, in remodeling the dendritic architecture of developing hippocampal neurons (2–25 DIV). Analysis of the dendritic architecture and the temporal and spatial expression patterns of the NMDARs and anchoring proteins in immature cultures revealed a strong positive correlation between synaptic expression of the NR2B subunit and dendritogenesis. With maturation, the pruning of dendritic branches was paralleled by a strong reduction in overall and synaptic expression of NR2B, and a significant elevation in synaptic expression of NR2A and PSD95. Using constructs that alter the synaptic composition, we found that either over-expression of NR2B or knock-down of PSD95 by shRNA-PSD95 augmented dendritogenesis in immature neurons. Reactivation of dendritogenesis could also be achieved in mature cultured neurons, but required both manipulations simultaneously, and was accompanied by increased dendritic clustering of NR2B. Our results indicate that the developmental increase in synaptic expression of PSD95 obstructs the synaptic clustering of NR2B-NMDARs, and thereby restricts reactivation of dendritic branching. Experiments with shRNA-PSD95 and chimeric NR2A/NR2B constructs further revealed that C-terminus of the NR2B subunit (tail) was sufficient to induce robust dendritic branching in mature hippocampal neurons, and suggest that the NR2B tail is important in recruiting calcium-dependent signaling proteins and scaffolding proteins necessary for dendritogenesis.

## Introduction

Before the dendritic arbor stabilizes in the mature CNS and dendritic spines are formed to allow communication between neurons, large-scale neuronal morphological changes occur during the first weeks of postnatal development that include growth of dendritic branches followed by elimination (pruning) of excessive and mis-targeted branches [1]. Accumulating evidence indicate that reduced synaptic connectivity, due to a diminished dendritic arbor complexity, plays an important role in the cognitive and memory impairment observed during aging and in psychiatric and neurodegenerative disorders [2]. Understanding thus the molecular mechanisms that underlie dendrite dynamics and stabilization during development might allow reactivation of dendritogenesis of mature neurons to enhance neuronal connectivity in older persons and patients with brain disorders.

Considerable evidence points to a role for NMDARs in regulating the neuronal architecture during early developmental stages [3]. Based on the strong correlation between structural alterations and the developmental switch from the NR2B to NR2A subunit, we argue that NR1NR2B receptors promote structural reorganization of connections, whereas NR1NR2A receptors facilitate stability [4]. Indeed, recent over-expression and knock-down studies indicate that the NR2B subunit of the NMDAR regulates dendritic branch formation and patterning *in vitro* [5] and *in vivo* [6]. However, the molecular underpinnings of NR2B-mediated structural remodeling are not well understood.

The NR2A and NR2B subunits are closely related, but the cytoplasmic C-terminal sequence of each is quite distinct at the level of amino acids and can influence interactions of the receptor with members of the family of membrane-associated guanylate kinases (MAGUKs), which include PSD-95, PSD-93, SAP97 and SAP102 [7]. These MAGUKs are essential for clustering and anchoring of NMDARs and other proteins at synapses [8]–[10]. Because of their high level of homology, and their capacity to compensate for one another [9], [11], determining specific binding partners of the different MAGUKs, and defining how they individually contribute to synapse and dendrite development, has been a challenge. However, we do know from comparative studies that PSD95 and SAP102 can have distinctly different binding partners, spine distribution, mobility, and temporal expression patterns [4], [10], [12]–[14]; but see [15]). These and other studies led us to hypothesize that synaptic insertion of PSD95/NR2A-NMDARs complexes during development displaces SAP102/NR2B-NMDARs complexes, and thereby limits plasticity [4].

Here we examine the role of the NR2A and NR2B subunits, and the MAGUKs PSD95 and SAP102, in remodeling the dendritic architecture of developing hippocampal neurons (2–25 DIV). We first establish the sequential alterations in dendritic architecture, and the spatial and temporal expression patterns of each protein: we find a strong positive correlation between synaptic expression of the NR2B subunit and dendritogenesis. We also show that with maturation, when the synaptic expression of PSD95 and NR2A dominate, dendritic branching is suppressed. We provide direct evidence that PSD95 limits dendritogenesis by occluding clustering of NR2B-NMDARs. Use of NMDAR chimera constructs further indicates that the C-terminal of NR2B (tail) is critical in dendritogenesis.

## Results

### Developmental changes of neuronal architecture in long-term hippocampal cultures

**Figure 1:**
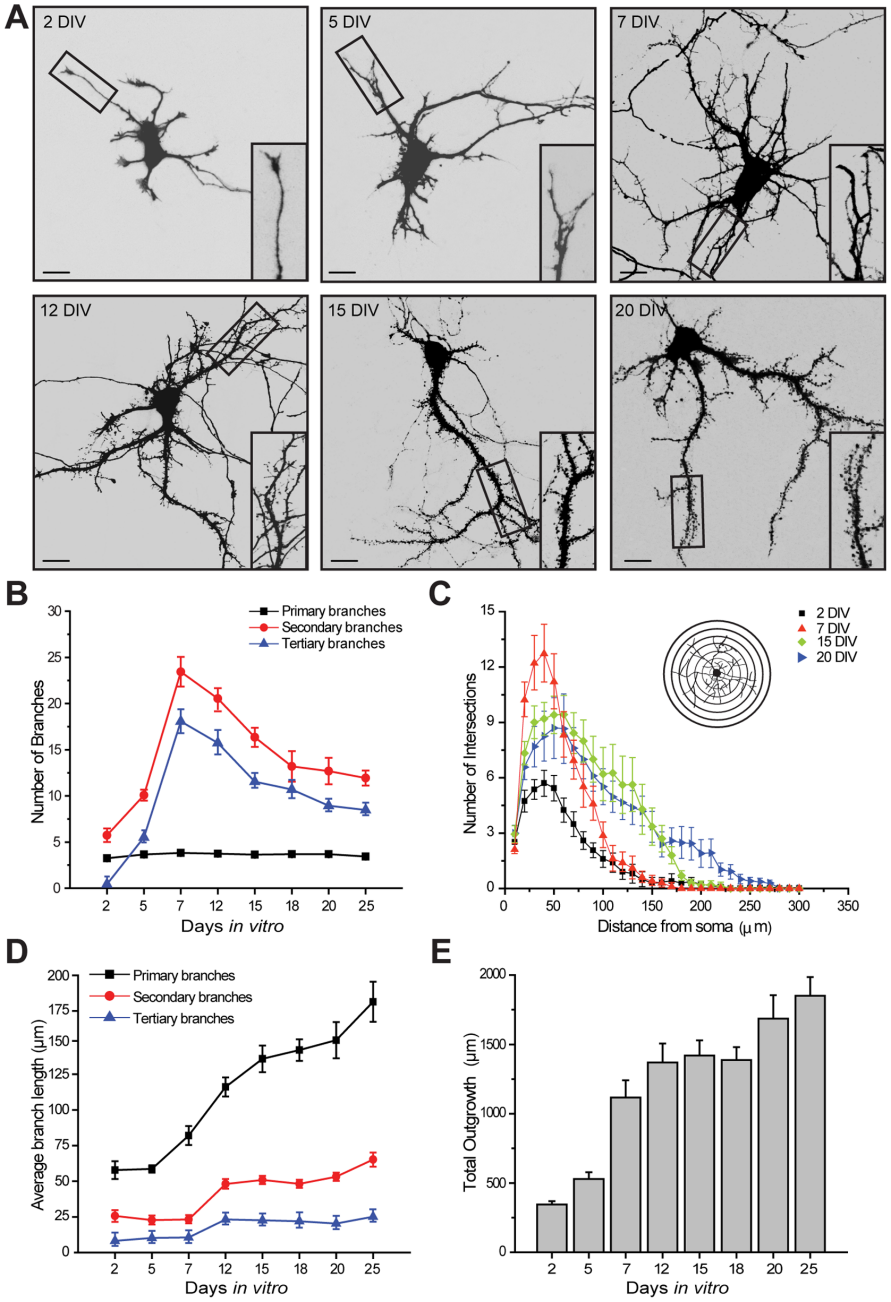
Changes in dendritic architecture during hippocampal development *in vitro*. ***A***, Cultured hippocampal neurons were transfected with GFP at 0 DIV with Amaxa nucleofection and fixed at 2, 5, 7, 12, 15 and 20 DIV. Images of representative GFP-expressing hippocampal neurons at the different stages are shown. Scale bar is 25 μm. Inset: Magnified views of boxed areas showing examples of dendritic branches. Note that dendritic arborization increases until 7 DIV and then gradually, but profoundly, drops during the next 2 weeks. Also note that whereas immature neurons display filopodia-like structures, mature neurons are predominantly decorated by dendritic spines. ***B***, Quantification of the average number of primary, secondary or tertiary dendritic branches of neurons expressing GFP at the developmental stages indicated in the graph. ***C***, Sholl analysis of neurons at different stages of development documents that cells at 7 DIV display an increased number of intersections close to the soma (25–50 μm). ***D–E***, Averaged length of primary, secondary and tertiary branches (***D***) and total outgrowth (***E***) of hippocampal neurons at different developmental stages. At least 20 neurons, obtained from 3 independent experiments, were analyzed for each stage. Figures show Mean ± SEM.

To elucidate the mechanisms that limit dendritogenesis with maturation, we first tracked the development of the normal dendritic architecture in maturing hippocampal neurons. Amaxa nucleofection was used to electroporate cultured hippocampal neurons with a plasmid encoding a fluorescent protein (GFP) at 0 DIV. Two to twenty five days later, neurons were fixed and their primary, secondary and tertiary branches were quantified. Large scale structural changes were seen in neurons during development (Figure 1): the number of secondary and tertiary dendritic branches strongly increases during the first 7 DIV and then gradually but profoundly drops over the next 2 weeks in culture (Figure 1A–B). It can also be observed that filopodia-like structures are prominent in immature neurons, but disappear with development, and are replaced by dendritic spines that are characteristic of mature neurons (Figure 1A, insets). The number of primary dendrites does not change with maturation (Figure 1A–B). Because in all our experiments the number of primary branches remains unaltered, this parameter is no longer presented.

Sholl analysis was used to determine the distribution of branches across the entire developing dendritic tree by counting the number of branches that cross a set of concentric circles drawn around the cell soma at increasing radial distances (Figure 1C, *inset*; 10-μm Sholl bins). Results are shown for neurons at 2, 7, 15 and 20 DIV. The analysis revealed that the number of branches localized close to the soma (25–50 μm) peaks at 7 DIV, but these branches largely disappear by 15 and 20 DIV (Figure 1C), suggesting that the newly generated branches of immature neurons are pruned with maturation. Sholl analysis also documented that with development, the branch density increases with distance from the soma (Figure 1C), a result that correlates with an approximately three-fold increase in the average length of the primary branches with maturation (Figure 1D). Consistent with this robust increase in the length of primary processes, the total outgrowth of hippocampal neurons markedly increases during the first three weeks of development *in vitro*, and stabilizes thereafter (Figure 1E).

Collectively, in agreement with previous studies *in vitro* [16] and *in vivo* [6], [17] the above quantitative data reveal marked maturational changes in the dendritic architecture of cultured hippocampal neurons; secondary and tertiary dendritic branches are robustly generated during the first week of development, but then are largely pruned. In contrast, the number of primary dendrites remains constant, although their increased dendritic length has a profound impact on neuronal geometry.

### Over-expression of NR2B does not promote dendritic branching in mature hippocampal neurons

**Figure 2:**
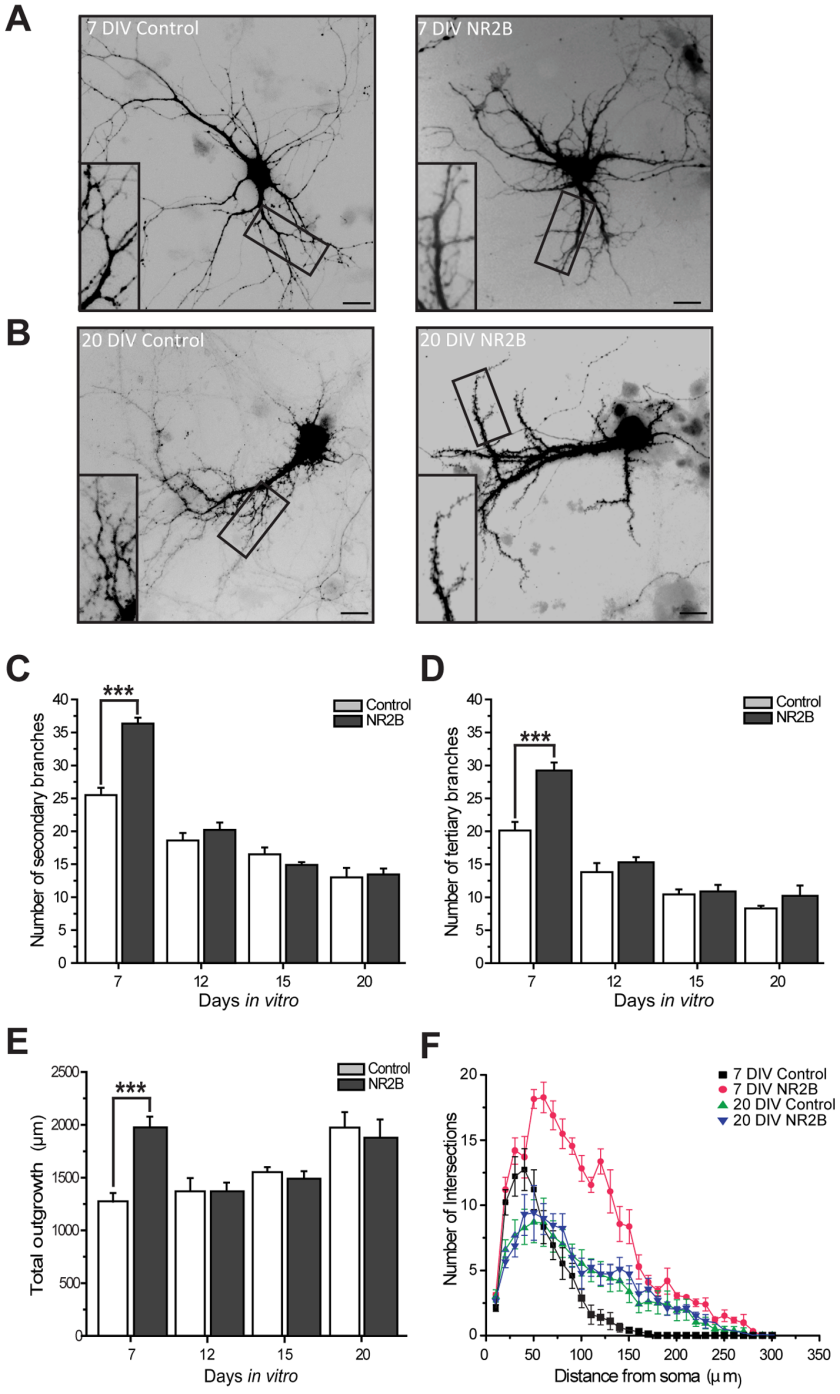
Over-expression of NR2B in hippocampal neurons promotes dendritic branching only in immature cells. Cultured hippocampal neurons were transfected with GFP alone (control) or with NR2B (NR2B) and fixed at 7, 12, 15 and 20 DIV. To express GFP and/or NR2B in neurons for 3 days, neurons were transfected with either CaPO_4_ (<12 DIV) or magnetofection-based methods (>12 DIV). ***A–B***, Images of representative control hippocampal neurons or expressing NR2B at 7 DIV (***A***) or 20 DIV (***B***). Scale bar is 25 μm. Note that NR2B expression induces branching only at 7 DIV, while in hippocampal neurons at 20 DIV NR2B expression results in a dendritic architecture that is similar to that of controls. ***C–E***, Averaged length of secondary (***C***) and tertiary (***D***) branches, and total outgrowth (***E***) of hippocampal neurons expressing GFP alone or GFP plus NR2B at different developmental stages. ***F***, Sholl analysis of control neurons or NR2B-expressing neurons at 7 DIV and 20 DIV. For each developmental stage and condition, at least 20 neurons, obtained from 3 independent experiments, were analyzed. Figures show Mean ± SEM. *** p<0.001 (ANOVA).

Given that the NR2B subunit of the NMDAR regulates dendritic branch formation and patterning *in vitro* [5] and *in vivo* [6], we expected that its reduced synaptic expression (as shown below) would account for the limited number of secondary and tertiary branches observed in more mature hippocampal neurons. We thus argued that boosting the expression of the NR2B subunit should lead to an increase in dendritic branch number. To test this hypothesis, we used CaPO_4_ (<12 DIV) or magnetofection-based transfection methods (>12 DIV) to transiently express GFP alone (control) or GFP plus NR2B in neurons for 3 days. Transfected cells were fixed at 7, 10, 15 and 20 DIV neurons, images taken and the neuronal morphology analyzed. As shown in Figure 2, the impact of NR2B over-expression on the dendritic architecture varied with the developmental stage of the hippocampal neurons. Immature neurons (7 DIV) that over-expressed NR2B displayed a much more complex dendritic tree relative to control cells (Figure 2A), with ∼50% increases in the number of secondary (Figure 2C) and tertiary branches (Figure 2D); in contrast, over-expression of NR2B subunits had no detectable effects on the architecture of more mature hippocampal neurons (12, 15 and 20 DIV) (Figure 2B shows data for 20 DIV). In particular, no significant changes were detected in the number of secondary or tertiary branches (Figure 2C–D). Summation of the lengths of all dendritic branches also confirmed that over-expression of NR2B was only effective in changing the dendritic architecture of immature hippocampal neurons (Figure 2E). Sholl analysis further revealed that the NR2B-induced novel branches are expressed at distance of 50–150 μm from the neuronal soma (Figure 2F).

### Patterns of expression of NMDAR subunits and MAGUKs during development

**Figure 3:**
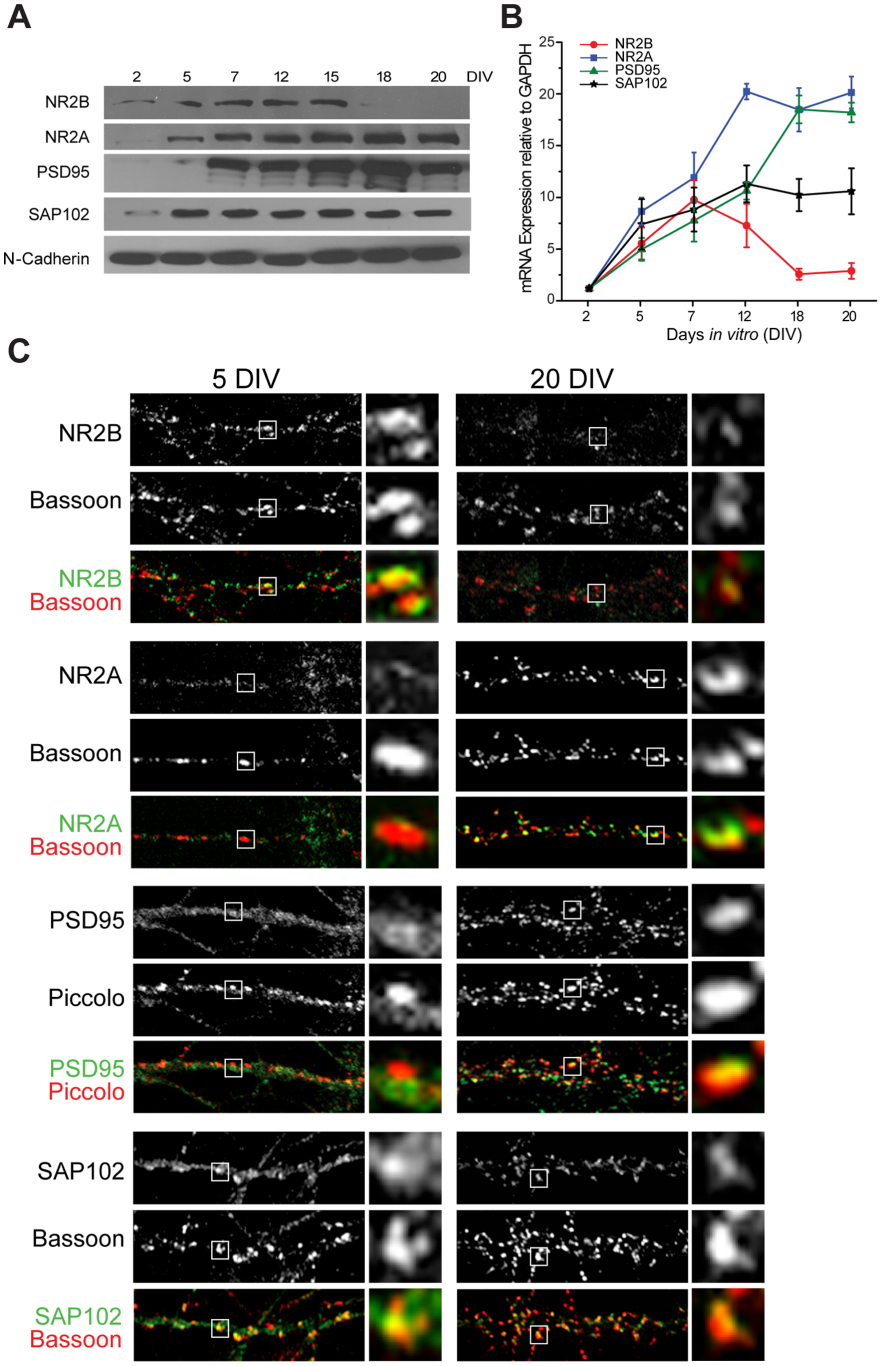
Expression of NMDAR subunits NR2A and NR2B, and MAGUKs PSD95 and SAP102, at different stages of development. ***A***, Total protein extracts were obtained from hippocampal neurons at 2, 5, 7, 12, 15, 18, and 20 DIV and immunoblotted with antibodies against NR2B, NR2A, PSD95, SAP102, or N-cadherin as loading control. ***B***, Results of qRT-PCR run with primers designed to determine mRNA levels for NR2B, NR2A, PSD95, or SAP102 at the same developmental stages; results were normalized against levels of GAPDH mRNA. Note that NR2B expression decreases with development, while NR2A and PSD95 increases. Expression of SAP102 remains relatively stable at these developmental stages. ***C***, Immunodetection of endogenous NR2B, NR2A, PSD95, or SAP102 (green) and co-localization with presynaptic marker Bassoon or Piccolo (red) at 5 and 20 DIV. Insets show magnified views of boxed areas showing examples of synaptic clusters located in close apposition to presynaptic clusters.

We next asked why the expression of the NR2B subunit in mature neurons is unable to induce dendritogenesis. Earlier studies used findings from *in utero* electroporation and electrophysiology experiments to conclude that SAP102 mediates synaptic localization of NMDARs and AMPARs during early development and this role is subsumed by PSD95 in mature neurons [10]. It is also shown that during development, and in activity-dependent manner, PSD95 regulates the trafficking of NR2A-NMDARs towards the postsynaptic density of spines, which hereby displaces synaptic NR2B-NMDARs [10], [13], [18]. These results support our hypothesis that, as maturation proceeds, PSD95 (which anchors NR2A-rich receptors) displaces SAP102, which then translocates along with its coupled NR2B-rich receptors from the postsynaptic membrane towards the extra-synaptic membrane [4]. To gain further insight into this process, we first examined the temporal and spatial expression patterns of the NR2A, NR2B, PSD95 and SAP102 proteins at several stages of hippocampal neuronal development (2–20 DIV). Western blotting of total protein extracts (Figure 3A) shows that all proteins are present in these cultures; however, their expression patterns robustly change with time. NR2B is present in 2 DIV hippocampal cultures, gradually increases with development, peaks at 15 DIV, and then abruptly declines. In contrast, while the expression of NR2A and PSD95 is not reliable detectable in 2 DIV immature neurons, their protein bands gradually become more intense over development, and peak in mature neurons (Figure 3A). Expression of SAP102 is detected at very early developmental stages (2 DIV) and remains high throughout the maturation of hippocampal cultures (Figure 3A).

Quantitative real-time reverse transcriptase PCR (qRT-PCR) analyses show that the pattern of mRNA expression for NR2B parallels its biphasic protein expression level during hippocampal culture development (Figure 3B). The increases in mRNA levels for NR2A, PSD95 and SAP102 also match their protein expression levels in maturing hippocampal cultures (Figure 3B).

Next we performed immunofluorescent staining to characterize the developmental expression and surface localization of endogenous NR2B and NR2A subunits and of their anchoring proteins PSD95 and SAP102 in hippocampal neurons at 5 and 20 DIV. Double staining with the presynaptic markers Bassoon or Piccolo was carried out to visualize the synaptic enrichment of NMDARs and MAGUKs. As shown in Figures 3C, the localization of these proteins largely depends on the stage of maturation of hippocampal cultures. At 5 DIV, NR2B-immunoreactive (IR) clusters are observed in close apposition to Bassoon-positive puncta, suggesting a synaptic distribution of this NMDAR subunit. By contrast, NR2B-IR is very low and diffuse at 20 DIV, and clusters are only detected sporadically. We also found that at 5 DIV the distribution of NR2A and PSD95 is diffuse, with scarce co-localization with presynaptic puncta. Conversely and as expected, neurons at 20 DIV showed high density of intense NR2A- and PSD95-IR puncta in close proximity to presynaptic protein markers, indicating a synaptic localization of both proteins. SAP102-IR is observed in close apposition to Bassoon-positive puncta at 5 DIV and maintain clearly visible in mature neurons.

### PSD95 inhibits dendritic clustering of NR2B-NMDARs and limits dendritogenesis

**Figure 4:**
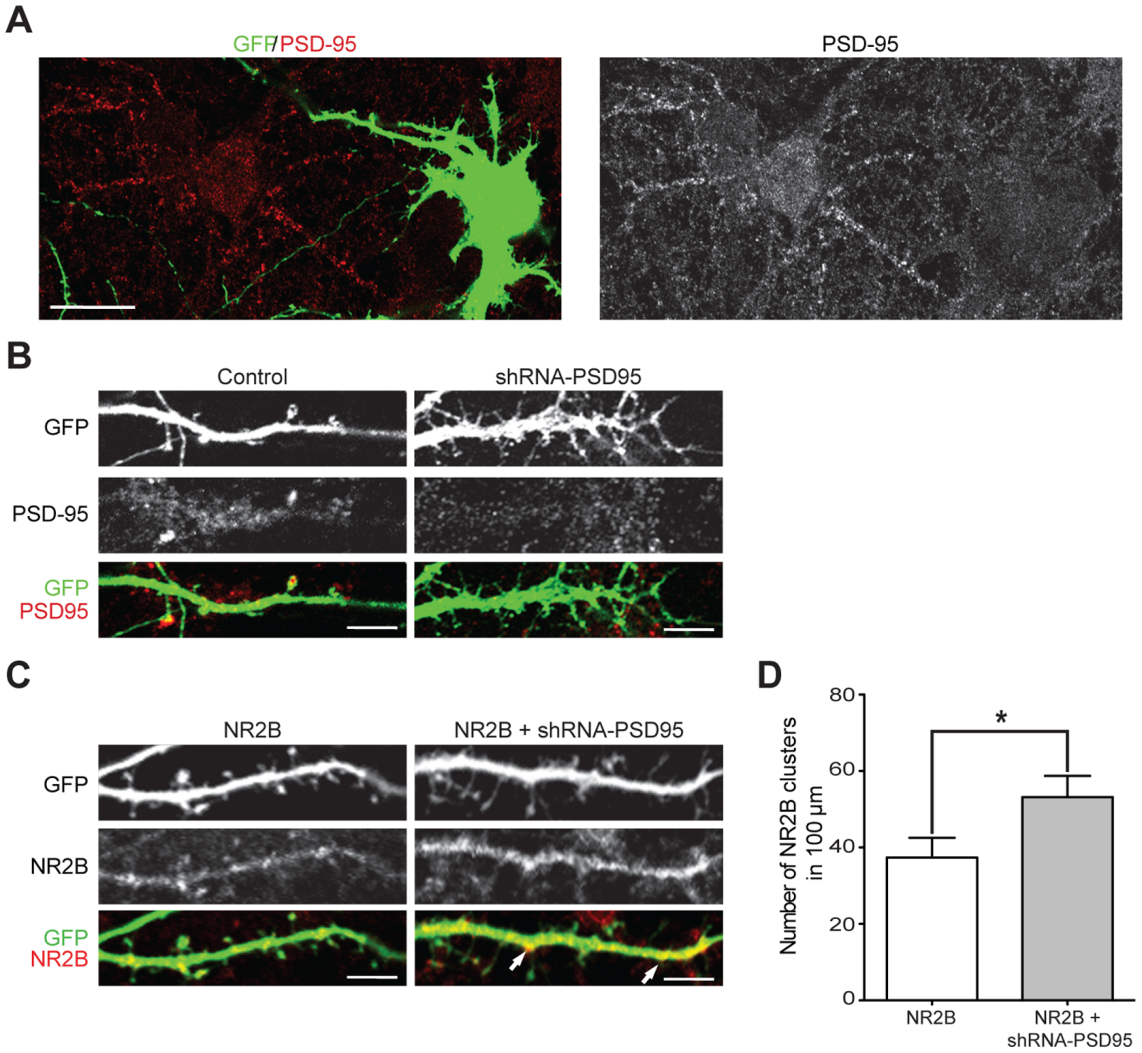
Knockdown of PSD95 promote NR2B clustering in mature hippocampal neurons. ***A***, Cultured hippocampal neurons were transfected with a magnetofection-based method at 15 DIV with GFP plus shRNA-PSD95. At 20 DIV, cultures were fixed and stained with anti-PSD95 antibody. Confocal images (one optical section of 1 μm) show transfected cell (green) and PSD95-IR (red). Scale bar: 20 μm. ***B***, Representative images of neuronal branches expressing GFP alone (Control; left images) or GFP plus shRNA-PSD95 (shRNA-PSD95; right images) and immunostained against PSD95 (red). Note that shRNA-PSD95 causes a significant loss of mature spines and an increase in the number of filopodia-like structures. Images were constructed from a stack of optical section (total 1.5 μm). Scale bar: 5 μm. ***C***, Representative images of neuronal branches expressing GFP plus NR2B (NR2B; left images) or GFP plus NR2B and shRNA-PSD95 (NR2B+shRNA-PSD95; right images) and immunostained against NR2B (red). Images were constructed from a stack of optical section (total 1.5 μm). Scale bar: 5 μm. ***D***, Quantification of total NR2B cluster number per 100 μm of neurite length, in NR2B neurons or those expressing both NR2B and shRNA-PSD95. For each condition at least 9 neurons were analyzed. Figures show Mean ± SEM. * p<0.05 (t-test).

To determine whether changes in synaptic PSD95 levels are an important component of the mechanisms that underlie NR2B-NMDAR-dependent dendritogenesis, we knocked-down the expression of this MAGUK in 15 DIV hippocampal neurons and then examined the clustering of the NR2B subunit and changes in neuronal architecture at 20–21 DIV. We used previously characterized plasmid-based RNA interference (shRNA-PSD95) [19] to reduce the expression of PSD95. In agreement with previous studies [19], [20], shRNA-PSD95 resulted in a robust reduction of PSD95 expression (Figure 4A) and a significant loss of mature spines and an increase in the number of shaft synapses and filopodia-like structures (Figure 4B). Scrambled shRNA was used as a control and found to be ineffective (not shown). To address the specificity of shRNA-PSD95 and discard effect on other MAGUK proteins, western blot and qRT-PCR experiments were performed to evaluate the expression of SAP102, PSD93, and SAP97. We found that the expression of none of these MAGUKS was affected by knocking-down PSD95 expression (Supplementary Figure 1 in File S1).

To assess whether PSD95 negatively controls dendritic branching in mature neurons by occluding the synaptic insertion of NR2B-NMDARs, we first analyzed the effects of PSD95 knock-down on the distribution of the endogenous NR2B subunit. Hippocampal cultures (15–16 DIV) were transfected with GFP alone (control) or with GFP plus shRNA-PSD95, fixed on 20–21 DIV, and assayed immunocytochemically with use of an antibody against NR2B. Mature neurons displayed very low levels of endogenous NR2B-IR; staining was distributed diffusely and expression of shRNA-PSD95 did not significantly increase the clustering of NR2B (data not shown); this apparent discrepancy, however, may be explained by our observation that endogenous expression of the NR2B subunit is very limited in mature neurons (Figure 3). We next transfected 15–16 DIV hippocampal cultures with GFP and NR2B (hereafter NR2B) or GFP plus both NR2B and shRNA-PSD95 (hereafter NR2B+shRNA-PSD95), fixed the cells at 20–21 DIV, and analyzed the distribution of the NR2B subunit (endogenous and exogenous). Cells transfected with the NR2B subunit displayed few NR2B-IR clusters that were distributed throughout the dendrite and sometime located in dendritic spines (Figure 4C). We found that neurons that expressed NR2B+shRNA-PSD95 presented a significant increase in the number of NR2B-IR clusters compared to neurons transfected with NR2B alone (Figure 4C–D); note that NR2B+shRNA-PSD95-expressing neurons contain few spines and that NR2B-IR clusters are presumably located at shaft synapses.

**Figure 5:**
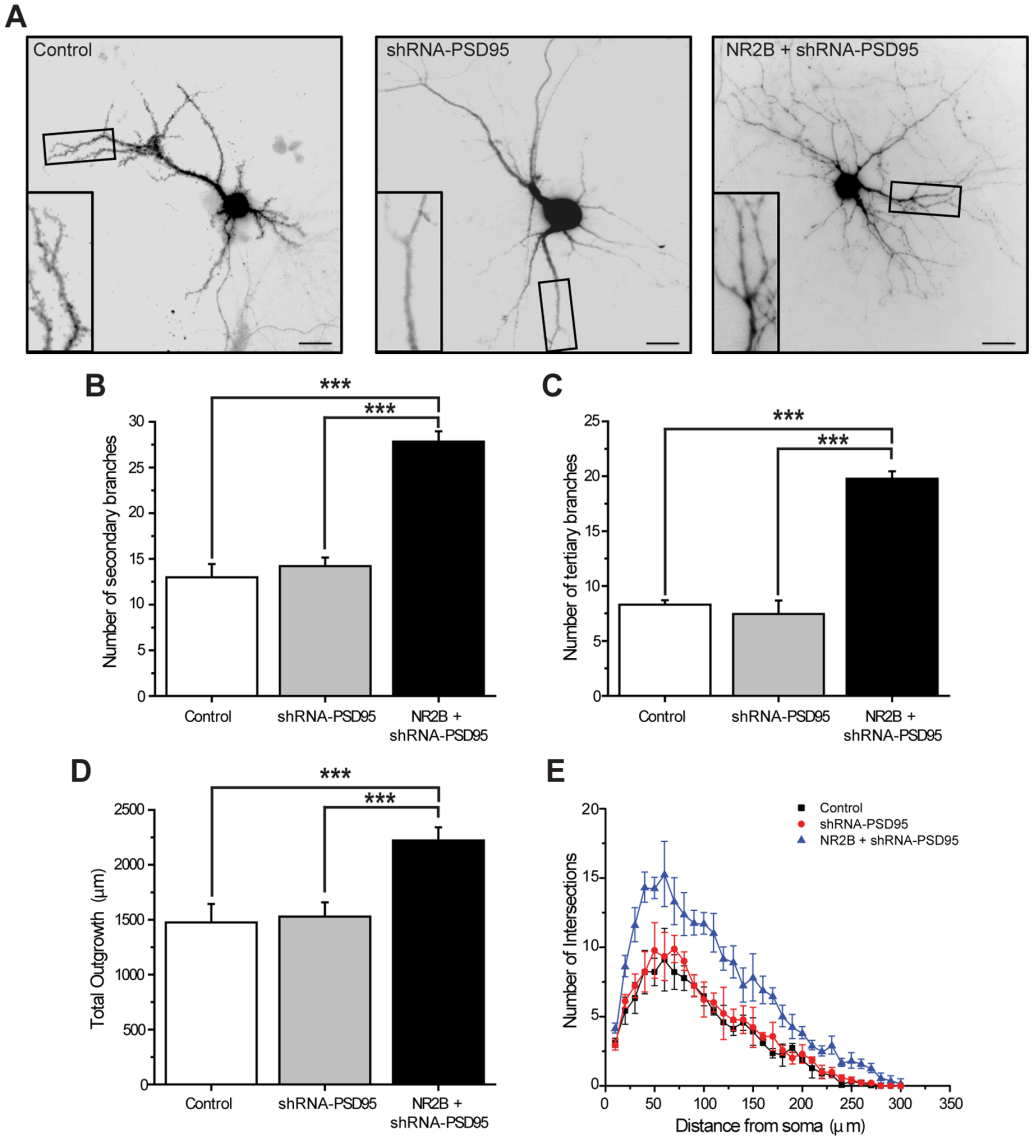
Simultaneous over-expression of NR2B and knockdown of PSD95 induce dendritic branching in mature hippocampal neurons. ***A***, Cultured hippocampal neurons were transfected with a magnetofection-based method at 15 DIV with GFP alone (control; left image), GFP plus shRNA-PSD95 (shRNA-PSD95; middle image), or GFP plus NR2B and shRNA-PSD95 (NR2B+shRNA-PSD95; right image). At 20 DIV, cultures were fixed and images taken. Scale bar is 25 μm. Inset: Magnified views of boxed areas showing examples of dendritic branches. Note that neurons that express NR2B+shRNA-PSD95 display a more complex dendritic architecture compared to control or shRNA-PSD95-expressing cells. ***B–C***, Quantification of the average number of secondary (***B***) and tertiary (***C*** **)** processes: neurons expressing NR2B+shRNA-PSD95 have more branches relative to control neurons and to cells transfected with shRNA-PSD95. ***D–E***, Total outgrowth (***D***) and Sholl analysis (***E***) of hippocampal neurons expressing the different constructs as indicated. For each condition, at least 20 neurons, obtained from 3 independent experiments, were analyzed. Figures show Mean ± SEM. *** p<0.001 (ANOVA).

To establish whether PSD95 negatively controls dendritic branching in mature neurons, we analyzed the dendritic architecture of 21 DIV hippocampal neurons that were transfected at 15 DIV with GFP alone (control), GFP plus shRNA-PSD95 (shRNA-PSD95), or GFP plus NR2B and shRNA-PSD95 (NR2B+shRNA-PSD95). Figures 5A documents that simultaneous over-expression of NR2B and knock-down of PSD95 has a marked impact on dendritic branching with an approximately two-fold increase in the number of secondary branches (Figure 5B) and an approximately three-fold increase in the number of tertiary branches (Figure 5C). To further understand the nature of the morphological changes, we analyzed branch lengths. Averaged lengths of the primary, secondary, and tertiary branches were not significantly different between control neurons and neurons that expressed shRNA-PSD95 alone, or NR2B+shRNA-PSD95 (data not shown). However, due to increases in the number of secondary and tertiary branches, neurons expressing NR2B+shRNA-PSD95 displayed a significant increase in total dendritic outgrowth (Figure 5D). Sholl analysis revealed that (relative to control cells and those expressing shRNA-PSD95 alone) the density of branches was higher in neurons that expressed NR2B plus shRNA-PSD95, and this increase was independent of the distance of the branches from the soma (Figure 5E). In an additional control experiment, we show that transfection of neurons with NR2B+shRNA-PSD95 together with a plasmid coding for PSD95 counteracts the knock-down of PSD95, hereby avoiding changes in arborization (Supplementary Figure 2 in File S1).

These results indicate that the dominant synaptic expression of PSD95 and the limited synaptic expression of NR2B are key contributors to the restricted growth of dendritic arbors in mature cultured hippocampal neurons. Treatment of cultures with antagonists for the different receptors further show that activity mediated by NMDARs, and to a lesser extent by AMPARs, is required for the dendritogenesis mediated NR2B plus shRNA-PSD95 (Supplementary Figure 3 in File S1).

### C-terminal domain of NR2B is required to promote dendritic branching

**Figure 6:**
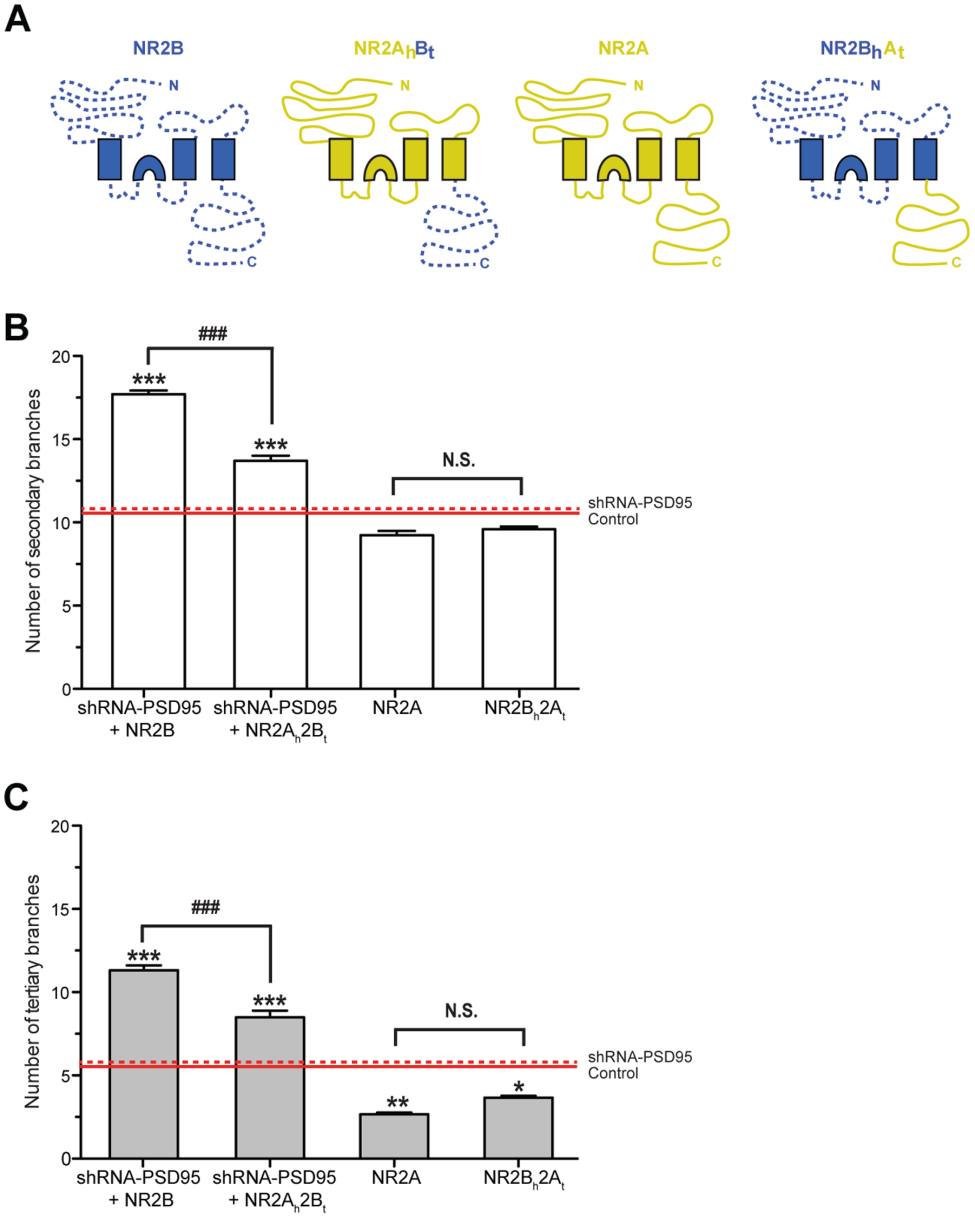
The C-terminal domain of NR2B is required to promote dendritic branching. ***A***, Schematic representation of a wild-type NR2B subunit (NR2B), the chimera NR2A_head_B_tail_ (NR2A_h_B_t_), a wild-type NR2A subunit (NR2A), and the chimera NR2B_head_A_tail_ (NR2B_h_A_t_). ***B–C***, Cultured hippocampal neurons were transfected with a magnetofection-based method at 15 DIV with GFP and the different wild-type and chimeric NR2 constructs, as indicated. As controls, cells were transfected with GFP alone (control; red continuous lines) or GFP plus shRNA-PSD95 (shPSD95; red dotted lines). For transfection with constructs that contain the NR2B tail (NR2B and NR2A_h_B_t_), PSD95 was knocked-down to allow clustering of these NR subunits. At 20 DIV, cultures were fixed, images taken and average number of secondary (**B**) and tertiary (**C)** dendritic branches was quantified. Note that in hippocampal neurons, the expression of a NR2 construct containing the C-terminal of the NR2B is a prerequisite for an increase in the number secondary and tertiary dendritic branches relative to control and shPSD95 conditions. For each condition, at least 20 neurons, obtained from 3 independent experiments, were analyzed. Figures show Mean ± SEM. * p<0.05, ** p<0.01, and *** p<0.001 (ANOVA) for branches relative to control conditions (control or shRNA-PSD95); and ### p<0.001 (t-test) for branches between the indicated conditions. N.S.  =  not significantly different.

We next molecularly dissected the NR2A and NR2B subunits to determine why NR1NR2B receptors are capable to induce dendritogenesis in mature neurons, but NR1NR2A receptors are not. Recent studies show that the cytoplasmic tail of the NR2B, but not the cytoplasmic tail of the NR2A subunit, plays a critical role in inducing LTP and synaptogenesis in hippocampal neurons [21], [22]. To establish the role of the N-terminal head *versus* the C-terminal tail of NR2A and NR2B subunits in dendritogenesis, we used the chimeric subunits NR2A_head_B_tail_ and NR2B_head_A_tail_ [21]: in these chimeras, i) the external N-terminal and transmembrane pore sequence of NR2A (NR2A_head_) is coupled to the cytoplasmic tail of NR2B (NR2B_tail_), and ii) the external N-terminal and channel-forming transmembrane pore sequence of NR2B (NR2B_head_) is coupled to the cytoplasmic tail of NR2A (NR2A_tail_) (Figure 6A). We found that co-expression of shRNA-PSD95 plus NR2A_head_B_tail_ in 21 DIV hippocampal neurons has a robust impact on the dendritic architecture with significant increases in the number of secondary and tertiary branches relative to control neurons (red continuous lines) and neurons expressing shRNA-PSD95 alone (red dotted lines) (Figure 6B). Our findings that those hippocampal neurons transfected with shRNA-PSD95 plus NR2B displayed a more complex dendritic architecture relative to those neurons expressing shRNA-PSD95 plus NR2A_head_B_tail_ (shown in Figure 6B-C as ###) further indicate that the different biophysical properties and/or kinetics mediated by the external N-terminal and channel-forming transmembrane pore sequence of the NR2A subunit also contribute to altering the dendritic arbor. To investigate the influence of the cytoplasmic tail of NR2A on the dendritic architecture, we transfected hippocampal neurons with NR2B_head_A_tail_ and used NR2A as internal controls: we omitted transfection with shRNA-PSD95, as this MAGUK is required to anchor the NR2A-NMDARs by its C-terminal into the postsynaptic membrane [4], [10]. Neurons that expressed either NR2B_head_A_tail_ or NR2A were found to display both a simple dendritic architecture relatively to control cells (Figure 6B-C). Using spinal cord neurons which lack NR2 NMDAR subunits [5], we observed similar results with the different chimeric constructs as in hippocampal neurons (Supplementary Figure 4 in File S1).

## Discussion

We provide evidence that developmental increases in synaptic expression of PSD95 obstruct the clustering of NR2B-NMDARs at postsynaptic sites, thereby restricting the reactivation of dendritic branching. We show that knock-down of PSD95 results in increased clustering of exogenous NR2B, and restores the dendritic branching capacity of mature neurons to a state that is normally seen only in early development (7 DIV). And finally, our results indicate that the cytoplasmic tail of NR2B-NMDARs is a prerequisite for the reactivation of dendritogenesis in mature hippocampal neurons.

We demonstrate that, concurrent with strong overall and synaptic expression of the NR2B subunit, large scale structural changes occur in the dendrites of developing hippocampal cultures, with branch number peaking at 7 DIV. During the second week of development, dendritic branches are pruned, and in parallel, NR2B at the synapse is replaced by NR2A and PSD95. The spatial and temporal patterns of the NMDAR subunits and PSD95 fit those previously reported in developing hippocampal neurons *in vitro* and *in vivo* based on electrophysiological, biochemical, immunocytochemical and electron-microscopic analyses [10], [12], [23]–[25]. Furthermore, we show that knock-down of PSD95 in mature neurons results in clustering of the NR2B subunit, and thereby induces dendritogenesis. Together, these data support our hypothesis that synaptic insertion of PSD95/NR2A-NMDARs complexes during development displaces SAP102/NR2B-NMDARs complexes, and thereby limits plasticity of hippocampal connections [4]. Indeed several studies indicate that SAP102 preferentially binds to the NR2B subunit [12], [14], [20]. On the other hand, many studies involving PSD95 knock-down or knock-out show little or no change in the NMDAR component of synaptic transmission [11], [26]–[30]. The effect of reduction of PSD95 expression, however, depends on the developmental stage of the neurons and their synapses. Thus, juvenile hippocampal neurons (P15–P20), obtained from mice in which PSD95 was knocked-out [27] or knocked-down [10], display NMDAR-mediated currents with a larger contribution of the NR2B subunit than in their wild type controls; and recent studies that use electron microscopy tomography in mature hippocampal neurons also show that knock-down of PSD95 leads to patchy loss of post-synaptic density (PSD) at the synaptic periphery, where mostly AMPARs are located, whereas the central region of the PSD, where NMDAR-type structures tend to cluster, remains largely unaffected [31], [32].

Based on the above findings, we suggest the following scenario to explain the apparently contradictory findings on how PSD95 regulates the synaptic tethering of glutamate receptors: PSD95 anchors two populations of subsynaptic proteins that are located within the mature PSD, however, these proteins are differently affected by PSD95 knock-down. One population is resistant to PSD95 knock-down, and is centrally located at the PSD where PSD95 tethers NR2A-NMDARs; the other population is sensitive to PSD95 knock-down, and is located at the periphery of the PSD where PSD95 anchors AMPARs. When the level of PSD95 is reduced (with use of RNAi), the PSD95-AMPAR population is largely eliminated, and allows SAP102 proteins that are located relatively far (50-250 μm) from the postsynaptic membrane [14], to become closer to the membrane. In juvenile neurons, SAP102 will recruit NR2B subunits that are abundantly present at the extrasynaptic membrane leading to the presence of synaptic NR2B-NMDARs, which influences the decay-times of NMDAR currents; by contrast, in adult cultured neurons, the lack or limited expression of NR2B does not fill up the synaptic SAP102 slots and thereby not affect NMDAR-mediated transmission.

NR2B-dependent dendritogenesis could be mediated by the different biophysical properties and kinetics [33], and/or specific sequences in the C-terminal cytoplasmic tails of the NMDAR subunits, which in turn could recruit diverse types of molecules, such as signal transduction complexes, to the synapse [4], [5], [15], [34], [35]. We used pharmacological as well as a molecular approaches, to show that the dendritogenesis in mature hippocampal neurons requires both NMDAR channel activity and the C-terminal cytoplasmic tail of the NR2B subunit. Moreover, the observation that dendritic branching is reduced in cells expressing NR2A_head_NR2B_tail_ compared to WT-NR2B suggests that differences in channel biophysical properties and/or kinetics also contribute to dendritic arbor growth. These results thus suggest that a bipartite function of the NR2B subunit is a prerequisite for optimum dendritogenesis: the external domain and pore propagate NMDAR-mediated calcium influxes intracellularly, where they activate specific postsynaptic signaling complexes that are recruited and scaffolded by the receptor's tail. Recent data show that a direct binding between the NR2B C-terminal and RasGRF1 is required to induce dendritic branching in spinal cord and hippocampal neurons [5]. It remains to be determined whether other signaling proteins such as CaMKII (which directly binds to the NR2B tail and regulates synaptogenesis [22], [35] also contribute to dendritogenesis.

Knock-down of PSD95 alone (in the absence of exogenous NR2B) in adult neurons does not alter dendritic architecture (Figure 5): this is an intriguing finding for several reasons. First, over-expression of PSD95, in the absence or presence of D-APV and DNQX, in developing hippocampal neurons significantly reduces dendritogenesis ([36], [37] and data not shown). While it is possible that an artificial gain-of-function phenotype generated by over-expression of PSD95 is responsible for this effect on dendritic architecture, we also observed that PSD95 knock-down is only able to induce dendritic branching in immature neurons (<15 DIV) (data not shown). Thus, the lack of an effect of shRNA-PSD95 on the dendritic architecture in mature neurons may be related to the presence/absence of proteins that are key in the process of dendritogenesis (such as proteins that influence the neuronal cytoskeleton). A recent report by [38] also shows that PSD95 regulates dendritogenesis by directly interacting with the microtubule plus-end binding protein EB3, and that the co-localization of PSD95 and EB3 peaks at 12 DIV and is precipitously reduced thereafter. It is likely that the expression of proteins (other than EB3) that are known to regulate microtubule dynamics and dendritogenesis are also developmentally regulated and are absent in mature neurons [2]. Second, it is surprising that shRNA-PSD95 alone (in the absence of exogenous NR2B) in adult neurons does not affect the dendritic architecture, while it is well established that knock-down of PSD95 results in a patchy loss of PSD material in mature synapses [32], and profoundly affects spine morphology ([39]; Figure 4) as well as synaptic plasticity [19], [39]–[41]; these latter phenomena likely derive from the removal of PSD95-interacting proteins, such as excitatory neurotransmitter receptors, ion channels, signaling molecules, cytoskeletal elements, anchoring proteins, and adhesion molecules [42], [43]. The findings with knock-down of PSD95 indicate that synapse formation/maturation and dendritic arbor development are not necessarily concurrent phenomena [3]. It is plausible thus that PSD95-mediated microdomains are present in the PSD of mature neurons, and that their removal affects certain types of neuronal processes, including synaptogenesis and synaptic plasticity, but has little impact on the dendritic architecture. Our data support the idea that SAP102 (instead of PSD95) - which binds NMDARs via the NR2B subunit, AMPARs via stargazin, and a complex containing SynGAP [32], [44], [45] - forms another microdomain that is essential for dendritogenesis in mature neurons.

In sum, we favor a scenario where PSD95 at the PSD blocks the re-insertion of NR2B-NMDARs from the extrasynaptic membrane; in turn, the NR2B subunit uses its C-terminal tail sequences to recruit signaling proteins that propagate receptor-mediated intracellular calcium influxes and induce dendritogenesis. The limited expression of the NR2B subunit is also an essential factor in reactivating the growth of the dendritic arbor in mature hippocampal cultures.

## Experimental Procedures

### Neuronal cultures

All protocols involving rodents were carried out in accordance with NIH guidelines, and were approved by the Ethical and Bio-security Committees of Andrés Bello University and University of Concepción. Pregnant Sprague-Dawley rats were deeply anesthetized with CO2, decapitated and fetuses (6–12/pregnant rat) quickly removed and decapitated. For the preparation of ventral spinal cord cultures and hippocampal cultures fetuses of embryonic day (E) 14 and 18 we scarified, respectively [5], [37], [46]. Briefly, hippocampi and whole spinal cords were excised and placed into ice-cold HBSS (Thermo Scientific SH30268.01) containing 50 μg/ml penicillin/streptomycin (Life technologies 15070-063). For ventral spinal cord neurons the dorsal part of the cord was removed using a small razor blade. Hippocampi and spinal cords were minced and incubated for 20 min at 37°C in pre-warmed HBSS containing 0.25% trypsin (Life technologies 15090-046), then transferred to a 15 ml tube containing neuronal plating media [Spinal cord neurons: Minimum Essential Media (MEM) (Life technologies 11095-072) supplemented with 10% Horse serum (Hyclone SH30074.03), 1% L-glutamine (Life technologies 25030-081), 4 mg/mL DNase (Roche 04716728001); Hippocampal neurons: Dulbecco's modified Eagle's medium (DMEM) (Hyclone SH30081.02) supplemented with 10% horse serum (Hyclone SH30074.03), and 100 U/ml penicillin/streptomycin (Life technologies 15070-063)], cells were resuspended by mechanical agitation through fire-polished glass Pasteur pipettes of decreasing diameters. Cells were counted and plated (1×106 cells for morphological analyses and 20.000 cells for immunofluorescence) on freshly prepared poly-L-lysine-coated 6-well plates (1 mg/ml; 30.000-70.000 MW; Sigma P2636). Plating media was replaced by growth media [Spinal cord neurons: (70% MEM (Life technologies 11090-073), 25% Neurobasal media (Life technologies 21103-049), 1% N2 supplement (Life technologies 17502-048), 1% L-glutamine (Life technologies 25030-081), 1% penicillin-streptomycin (Life technologies 15070-063), 2% horse serum (Hyclone SH30074.03; lot AQH24495) and 1 mM pyruvate (Sigma); Hippocampal neurons: (Life technologies 21103-049) supplemented with B27 (Life technologies 17504044), 2 mM L-glutamine (Life technologies 25030-081), 100 U/ml penicillin/streptomycin (Life technologies 15070-063)]. On day 2, hippocampal neurons were treated with 2 μM cytosine arabinoside (AraC) for 24 hrs. Media was replaced every 3 days for all neurons.

### Transient Transfections

Transfection methodologies differed with cell type and developmental stage. At 0 DIV, hippocampal neurons were transfected with use of an Amaxa Rat Neuron Nucleofector Kit (LONZA, Allendale, NJ, USA), following manufacturer's instructions with some modifications, as previously described [37]. Briefly, hippocampal neurons were electroporated with use of the Amaxa Nucleofector Electroporation II Device (LONZA): 1 μg of plasmid coding for GFP was mixed with the transfection reagents for every 1×106 cells. The cells were plated in twenty four-well plates for morphological analyses. From 4–12 DIV, spinal cord and hippocampal neurons were transfected according to an adapted CaPO4 transfection protocol to reduce cell toxicity [5], [37], [47]. Briefly, after 1 hour of incubation in 5% CO2, cells were incubated for 20 min with transfection medium (pre-equilibrated in a 10% CO2 incubator to dissolve the DNA-CaPO4 precipitates), then returned to a 5% CO2 incubator until the morphology was analyzed. Hippocampal neurons (>12 DIV) were transfected with use of Neuromag transfection reagent (OZ Biosciences), according to recent publications [48], [49] as well as the manufacturer's instructions with slight modifications [37]. Thirty minutes before magnetofection, complete medium was replaced with serum-free Minimal Essential Medium (MEM). Plasmid DNA was incubated with Neuromag beads, in a ratio of 0.5∶1.75 μg DNA per μL nanobeads in 100 μL MEM for 15 minutes, and then was added drop wise to the cultures. Cells were incubated at 37°C on a magnetic plate (OZ Biosciences) for 15 minutes, and complete medium was restored after 1 hour. To maximize transfection efficiency, no more than 0.8 μg of DNA was transfected per well, in a 24 well plate.

Constructs used to transfect spinal cord and hippocampal neurons included: plasmids coding for GFP to visualize the neuronal morphology in detail; NR2A and NR2B which were kindly donated by Stefano Vicini [50]; a pSuper plasmid coding for shRNA-PSD95 and scrambled shRNA kindly donated by Morgan Sheng [19]; NR2A_head_B_tail_ and NR2B_head_A_tail_ chimeric subunits [21].

### Morphological analysis

Fluorescently labeled neurons were fixed in 4% paraformaldehyde and visualized with a confocal (Olympus FV 1000, Tokyo, Japan) or epi-fluorescence (Nikon eclipse Ti) microscope. The number of dendrites was counted and the length of each dendrite assessed as described [5], [36], [37], with use of the semi-automated Metamorph software (Universal Imaging). Images were contrast-enhanced to ensure that all branches were analyzed. To measure the length of individual dendrites, every branch segment arising from the soma was digitally marked from the origin of the branch to its termination. The investigator was blinded to the experimental conditions, to reduce researcher bias. Sholl analysis was performed with ImageJ (NIH), using a special plug-in designed by the Ghosh laboratory (available at http://www-biology.ucsd.edu/labs/ghosh/software/index.html).

### Western Blot

Whole cell lysates were prepared in a buffer containing 50 mM Tris HCL pH 7.4, 150 mM NaCl, 0.1% SDS, 1% NP-40, 0.5% sodium deoxycholate, plus proteinase and phosphatase inhibitors. Samples (20 μg) were run on a 10% SDS-PAGE gel, transferred to a nitrocellulose membrane, blocked with 5% milk in TBS, and incubated overnight at 4°C with primary antibodies against NR2A (1∶500, Life technologies, A-6473), NR2B (1∶500, Life technologies, A-6474), PSD95 (1∶10000, UC Davis/NIH NeuroMab Facility 75-028, USA), SAP102 (1∶2000; kindly donated by Johannes Hell), PSD93 (1∶1000, UC Davis/NIH NeuroMab Facility 75-057, USA), SAP97 (1∶1000, UC Davis/NIH NeuroMab Facility 75-030, USA), or N-Cadherin (1∶1000, Santa Cruz Biotechnology H-63, USA) (as loading control). Membranes were washed in TBS, incubated with an HRP-conjugated secondary antibody (1∶5000; Santa Cruz Biotechnology) for 1 hour at RT, washed, incubated with ECL solution (Perkin Elmer) for 1 min, and exposed for 1–3 min on Biorad films.

### Immunofluorescence

Immunofluorescences were performed as previously described [37], [51]. Briefly, Cells were rinsed twice in ice-cold PBS, fixed for 20 min in a freshly prepared solution of 4% paraformaldehyde in PBS, and washed 3 times with PBS; they were permeabilized for 5 min with 0.2% Triton X-100 in PBS and after several rinses in ice-cold PBS, were incubated in 1% BSA in PBS (blocking solution) for 30 min at room temperature, followed by an overnight incubation at 4°C with primary antibodies. Primary antibodies used were: Piccolo (1∶1000; polyclonal; Synaptic Systems, Germany), Bassoon antibody (1∶1000; monoclonal; Assay designs, Ann Arbor, MI, USA), NR2A (1∶250; polyclonal; Life technologies, A-6473), NR2B (1∶250; polyclonal; Life technologies, A-6474), PSD95 (1∶400; monoclonal; UC Davis/NIH NeuroMab Facility 75-028, USA), SAP102 (1∶250; polyclonal; kindly donated by Johannes Hell). Cells were washed 3 times with PBS, then incubated with Alexa-conjugated secondary antibodies (Life Technologies, USA) for 30 min at 37°C. Coverslips were mounted with Fluoromont-G (Electron Microscopy Sciences, Hatfield, PA, USA) and analyzed by confocal laser microscopy (Olympus FV 1000, Tokyo, Japan). Images were analyzed using NIH ImageJ software. Quantification of the density of IR-clusters was carried out under threshold conditions. Dual and triple color immunofluorescent images were captured by multitracking imaging of each channel independently, to eliminate possible cross-talk between the different fluorochromes. Co-localization was studied by superimposing all three color channels.

### Reverse transcriptase and quantitative real-time PCR (qRT-PCR)

Total RNA was extracted with TRIzol (Life technologies), according to the manufacturer's protocol. An equal amount of each sample (1–2 μg) was used for reverse transcription. qPCR was completed with use of Brilliant II SYBR Green QPCR Master Mix (Agilent technologies, 600828, USA). Data are presented as relative mRNA levels of the gene of interest normalized to GAPDH mRNA levels. Primers used were: NR2A Fw: TGGTCTATCAACGAGCAGTCA, Rv: CGATGAGCAGCATCACAAAC; NR2B Fw: TGCTGCAAGGGGTTCTGTAT, Rv: CAACCACCTCTGACCGTTCT; PSD95 Fw: GCCCTGTTTGATTACGACAA, Rv: ACTCGGTTCTGAGCTATGAG; SAP102 Fw: TTCCAGCCTGGGTTATCTTG, Rv: AAATGCCCTCTCCATCCTCT; SAP97 Fw: CCTTTCAAGTGCTGCTGTGA, Rv: CAGCACCTCATTTGGGACTT; PSD93 Fw: GGAGAGGGGAAATTCAGGTC, Rv: GCAGCACCTCCTGGAATAAT; GAPDH Fw: CATGGCCTTCCGTGTTCCTA, Rv: CCTGCTTCACCACCTTCTTGAT.

### Statistical Analyses

An ANOVA followed by the Dunnett post hoc test was used to detect intervals in which significant changes occurred for all data sets where a parameter was measured across time points within a treatment. Student's t-test was applied when two populations of responses were examined. In all figures, error bars represent the SEM; * p≤0.05, ** p≤0.01, *** p≤0.001.

## Supporting Information

File S1 (PDF)
