# Supplementary material for: PSD95 Suppresses Dendritic Arbor Development in Mature Hippocampal Neurons by Occluding the Clustering of NR2B-NMDA Receptors
Source: PLoS One. 2014 Apr 4;9(4):e94037. doi: 10.1371/journal.pone.0094037 (PMC3976375; doi:10.1371/journal.pone.0094037)
Supplement: File S1 — (PDF) [file pone.0094037.s001.pdf]

Supporting information

Supplementary Figure 1. Specificity of shRNA-PSD95 on expression of MAGUKs in mature hipocampal neurons. ***A-E,*** Hippocampal neurons were transfected with a magnetofection-based method at 15 DIV with GFP alone (control), or GFP plus shRNA-PSD95 (shRNA-PSD95). At 20 DIV, total protein extracts or total RNA were obtained. ***A***, Western blot analysis with use of antibodies against PSD95, SAP102, PSD93, or SAP97 to determine protein levels in hippocampal cultures. Use of antibody against N-Cadherin served as protein loading control. ***B-D***, qRT-PCR to measure mRNA levels for PSD95 (**B**), SAP102 (**C**), PSD93 (**D**), or SAP97 (**E**); results were normalized against mRNA levels of GAPDH. The results are representative of 3 independent experiments. Note that transfection with shRNA-PSD95 reduces specifically the expression of PSD95 and does not affect protein or mRNA levels of the other MAUKS. Figures show Mean ± SEM. *** $p<0.001$ (t-test).

Supplementary Figure 2. **Increased dendritogenesis in mature hippocampal neurons induced by expression of shRNA-PSD95 plus NR2B is mediated by specific PSD95 knockdown**. Hippocampal neurons were transfected with a magnetofection-based method at 15 DIV with either GFP alone (control), GFP plus NR2B and shRNA-PSD95 (NR2B+shRNA-PSD95), or GFP plus NR2B plus shRNA-PSD95 and PSD95 (NR2B+shRNA-PSD95+PSD95). At 20 DIV, cultures were fixed and images taken. ***A-B,*** Quantification of average number of secondary (**A**) and tertiary (**B**) dendritic branches on mature hippocampal neurons are shown. Note that neurons expressing NR2B+shRNA-PSD95+PSD95 display similar arborization to control neurons. For each condition, at least 20 neurons, obtained from 3 independent experiments, were analyzed. Figures show Mean ± SEM *** $p<0.001$ (ANOVA).

Supplementary Figure 3. **Functional NR2B-NMDARs are necessary to induce branching in mature hippocampal neurons.** ***A-B***, Cultured hippocampal neurons were transfected with a magnetofection-based method at 15 DIV with GFP alone (control) (**A**) or GFP plus NR2B and shRNA-PSD95 (NR2B+shRNA-PSD95) (**B**). The cultures were chronically treated, from the day of transfection (15 DIV), with D-APV (5 µM), ifenprodil (2 µM) or DNQX (1 µM). At 20 DIV, cultures were fixed and images taken. Quantification of average number of secondary (white bars) and tertiary (grey bars) dendritic branches on mature hippocampal neurons are shown. For each condition, at least 20 neurons, obtained from 3 independent experiments, were analyzed. Figures show Mean ± SEM. ** $p<0.01$ and *** $p<0.001$ (ANOVA) for secondary branches between control and NR2B+shRNA-PSD95 conditions; and ## $p<0.01$ and ### $p<0.001$ (ANOVA) for tertiary branches between control and NR2B+shRNA-PSD95 conditions.

Supplementary Figure 4. The C-terminal domain of NR2B is required to promote dendritic branching in spinal cord neurons. ***A-B,*** Cultured spinal cord neurons were transfected with calcium phosphate method at 4 DIV with GFP and the different wild-type and chimeric NR2 constructs, as indicated. At 10 DIV cultures were fixed, images taken and average number of secondary (**A**) and tertiary (**B**) dendritic branches was quantified. For each condition, at least 20 neurons, obtained from 3 independent experiments, were analyzed. Figures show Mean ± SEM. *** $p<0.001$ (ANOVA) for branches relative to NR2B; and §§§ $p<0.001$ (ANOVA) for branches relative to NR2A$_{head}$B$_{tail}$.

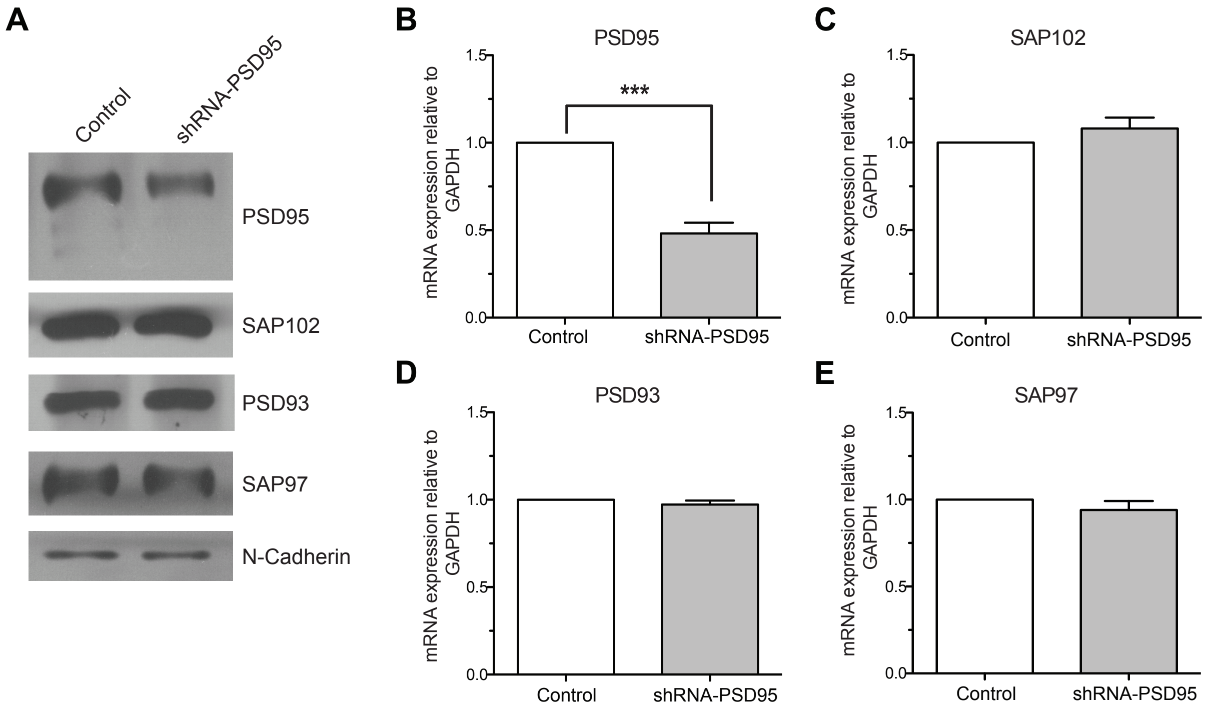

Supplementary Figure 1. Bustos et al., 2014

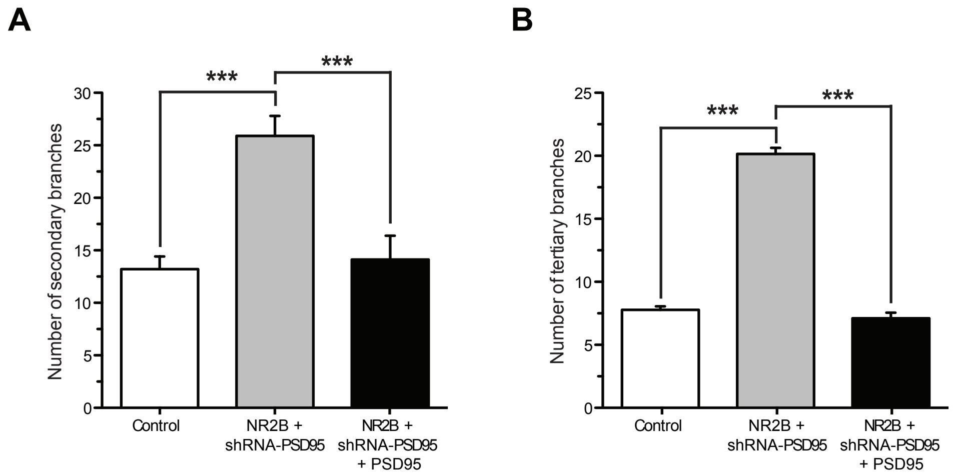

Supplementary Figure 2. Bustos et al., 2014

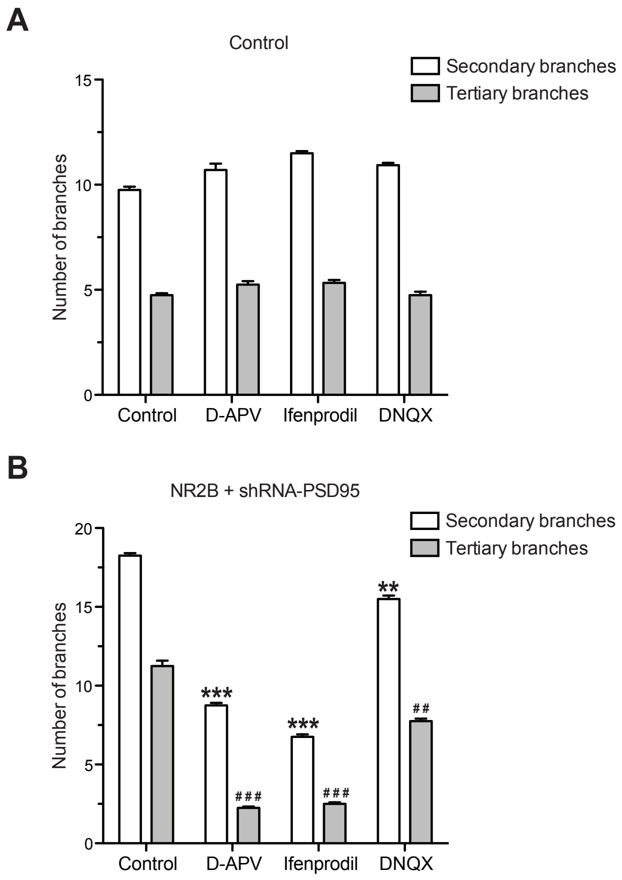

Supplementary Figure 3. Bustos et al., 2014

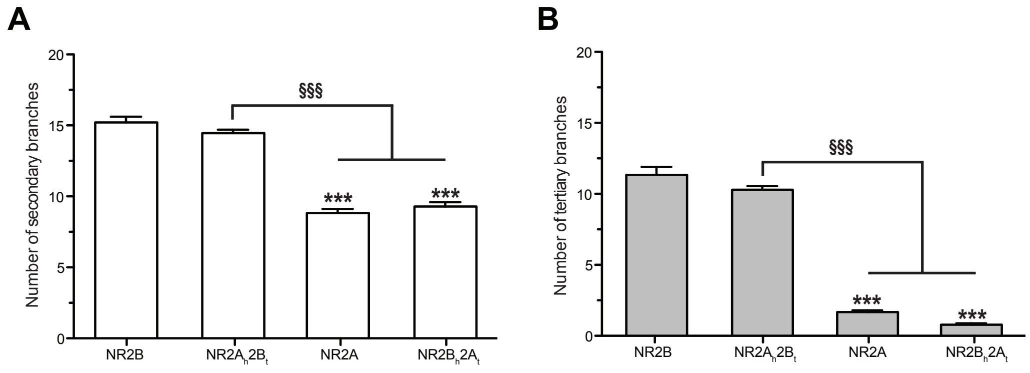

Supplementary Figure 4. Bustos et al., 2014
